# Deep Learning for Automated Detection and Localization of Traumatic Abdominal Solid Organ Injuries on CT Scans

**DOI:** 10.1007/s10278-024-01038-5

**Published:** 2024-02-16

**Authors:** Chi-Tung Cheng, Hou-Hsien Lin, Chih-Po Hsu, Huan-Wu Chen, Jen-Fu Huang, Chi-Hsun Hsieh, Chih-Yuan Fu, I-Fang Chung, Chien-Hung Liao

**Affiliations:** 1 Department of Trauma and Emergency Surgery, Chang Gung Memorial Hospital, Linkou, Chang Gung University, Taoyuan, Taiwan; 2 Department of Medical Imaging & Intervention, Chang Gung Memorial Hospital, Linkou, Chang Gung University, Taoyuan, Taiwan; 3 Institute of Biomedical Informatics, National Yang Ming Chiao Tung University, Taipei, Taiwan

**Keywords:** Deep learning, Computed tomography, Blunt abdominal trauma, Spleen injury, Liver injury, Renal injury, Artificial intelligence

## Abstract

Computed tomography (CT) is the most commonly used diagnostic modality for blunt abdominal trauma (BAT), significantly influencing management approaches. Deep learning models (DLMs) have shown great promise in enhancing various aspects of clinical practice. There is limited literature available on the use of DLMs specifically for trauma image evaluation. In this study, we developed a DLM aimed at detecting solid organ injuries to assist medical professionals in rapidly identifying life-threatening injuries. The study enrolled patients from a single trauma center who received abdominal CT scans between 2008 and 2017. Patients with spleen, liver, or kidney injury were categorized as the solid organ injury group, while others were considered negative cases. Only images acquired from the trauma center were enrolled. A subset of images acquired in the last year was designated as the test set, and the remaining images were utilized to train and validate the detection models. The performance of each model was assessed using metrics such as the area under the receiver operating characteristic curve (AUC), accuracy, sensitivity, specificity, positive predictive value, and negative predictive value based on the best Youden index operating point. The study developed the models using 1302 (87%) scans for training and tested them on 194 (13%) scans. The spleen injury model demonstrated an accuracy of 0.938 and a specificity of 0.952. The accuracy and specificity of the liver injury model were reported as 0.820 and 0.847, respectively. The kidney injury model showed an accuracy of 0.959 and a specificity of 0.989. We developed a DLM that can automate the detection of solid organ injuries by abdominal CT scans with acceptable diagnostic accuracy. It cannot replace the role of clinicians, but we can expect it to be a potential tool to accelerate the process of therapeutic decisions for trauma care.

## Background

Blunt abdominal trauma (BAT), resulting from incidents such as traffic crashes, falls, assaults, or occupational accidents, is a common occurrence in the trauma bay [1, 2]. Studies have reported a high prevalence of intra-abdominal injury following BAT, with rates ranging from 12 to 15% [3]. Among these injuries, the spleen, liver, and kidneys are the most frequently affected organs, constituting approximately 80% of all visceral injuries [4]. Since the 1980s, there has been a significant shift from surgical to nonoperative management (NOM) for BAT, with numerous studies demonstrating satisfactory outcomes [5–8]. The advancement of current diagnostic modalities, particularly computed tomography (CT), has played a crucial role in the NOM becoming a viable option for managing BAT patients [9]. CT scans provide accurate assessments of the severity of organ injury [10], hemoperitoneum, the presence of contrast extravasation [11], and viscus injury [12] and are crucial in predicting the need for prompt intervention [13], thereby making them the preferred diagnostic tool for hemodynamically stable patients. The extensive use of CT and a growing body of literature demonstrating promising results have led to the widespread acceptance of nonoperative management as the standard therapeutic strategy [14–17]. While CT and advanced technologies yield informative results, it is crucial for clinicians to possess the necessary skills to differentiate and detect abnormalities in high-resolution images. Although the CT image can present trauma or injuries, frontline clinicians might misdiagnose due to lack of experience, a crowded working environment, or overloading duty [18, 19]. Achieving higher diagnostic accuracy not only relies on the capabilities of the imaging modality but also on the clinician’s expertise.

The use of deep learning (DL) algorithms has proven capable of achieving diagnostic accuracy in medical imaging comparable to that of experts [20], whether applied to plain radiographs [21] or advanced medical images such as CT or magnetic resonance imaging (MRI) scans [22, 23]. As we enter an era characterized by collaboration between human expertise and computational power, DL algorithms hold the potential to revolutionize future medical practices, particularly by alleviating the workload of healthcare providers in emergency settings [24]. Despite these advancements, the availability of trauma-related algorithms to assist trauma surgeons in managing time-sensitive and life-threatening injuries is still limited [25–28]. Moreover, there is a clinical need for an explainable and transparent AI model to support emergency radiologists and clinicians, a need that remains unaddressed [29]. The ongoing development and implementation of specialized DL models in trauma care hold the potential to enhance patient outcomes further and support healthcare professionals in delivering swift and effective treatment. Previous studies of DL in torsal trauma imaging focused mainly on automatically grading specific organ injuries [30–32], injured area segmentation [22, 25], detecting active bleeding [33], and quantifying the hemorrhage amount in the chest and abdominopelvic CT scans [34–37]. The process of slice-level labeling in trauma imaging, particularly for deformed injured organs, demands extensive effort from specialists. Utilizing three-dimensional (3D) DL architectures presents an opportunity to employ scan-level labeling, significantly reducing the burden of labeling efforts [38]. Additionally, the advent of novel open-source frameworks for 3D organ segmentation offers promising avenues for expanded applications in CT imaging [39]. In the current study, we have developed a DL-based algorithm that combines an open-source segmentation model with a 3D classification network. This algorithm is designed to detect and diagnose visceral traumatic injuries, thereby assisting clinicians in handling these lethal injuries. To the best of our knowledge, at the time of submission, this is the first published approach to detect injuries across multiple organs.

## Materials and Methods

We selected patients from our trauma registry who underwent contrast-enhanced abdominal CT scans between May 2008 and December 2017 at Chang Gung Memorial Hospital, Linkou. The clinical information captured for each patient included age, gender, trauma mechanism, Abbreviated Injury Scale (AIS) scores for each body part, Injury Severity Score (ISS), interventions performed, final diagnosis, and outcome. We only enrolled the images acquired in our hospital. All of the images were acquired by the TOSHIBA Aquilion One 320 scanner in the emergency CT room. The protocols enrolled included routine abdominal scans, multiphase abdominal scans, and whole-body CT (only venous phase) with 5-mm slice thickness in axial view. In cases where a patient had multiple scans, only the earliest scan was included for analysis. We specifically utilized the venous phase of the scans for our study, excluding images of poor quality, those with artifacts, post-operative scans, or scans lacking an appropriate venous phase. CT scans obtained from external hospitals were not included in the study.

To ensure the accuracy of the image findings, a trauma surgeon who is an expert in medical image analysis with 13 years of clinical practice experience carefully reviewed images, original radiologist reports, trauma registry, and medical records to determine whether the patients had spleen, liver, or kidney injuries as scan level annotations. If the image or report is questionable, a senior radiologist with a trauma subspecialty was consulted to determine the true label. The grading of organ injuries was performed according to the AAST 2018 version [40], ensuring standardized and consistent assessment across all cases.

**Fig. 1:**
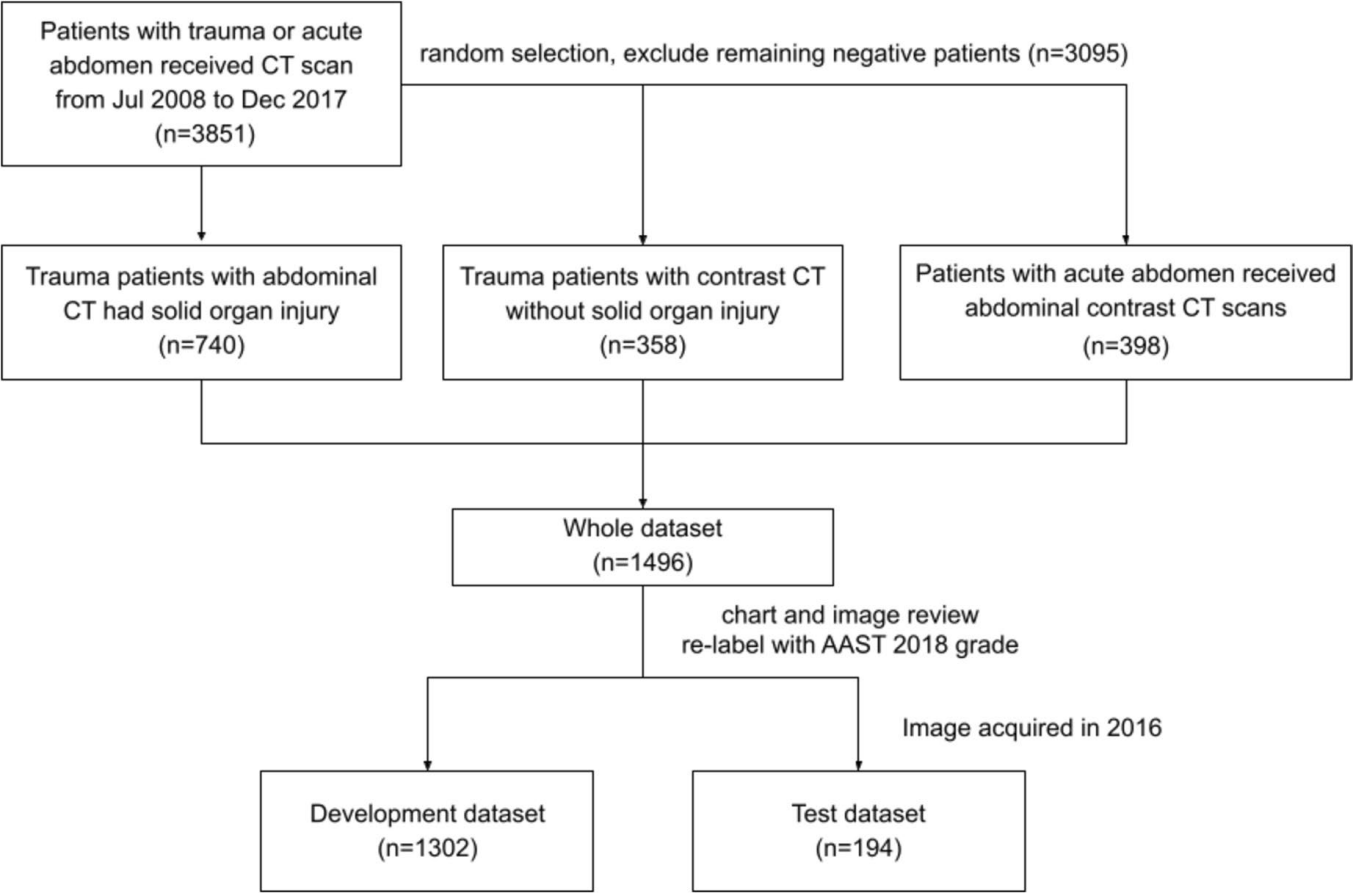
Dataset preparation

To increase the variability of abdominal CT scan images, we gathered an additional dataset from patients presenting with acute abdominal diseases such as appendicitis, biliary diseases, hollow organ perforation, intestinal obstruction, ischemic bowel, and other similar conditions, all of whom underwent abdominal CT scans in the emergency room. CT scans that depicted injuries to the spleen, liver, or kidneys were categorized as the positive group (indicating solid organ injury), while other findings were classified as the negative group (indicating nonsolid organ injury). Given the larger number of images in the negative group, we employed a random sampling method to balance the class distribution by selectively reducing the number of negative scans (Fig. 1).

To address potential selection bias, images acquired in the last year were set aside as an independent test set, while the remaining images were utilized as the developmental dataset. This approach ensures a more robust evaluation of the developed model’s performance on unseen data. This study was approved by the Institutional Review Board (IRB) of the Chang Gung Medical Foundation with No. 202002333B0.

### Image Preprocessing

The initial step involved obtaining the original CT scans in Digital Imaging and Communications in Medicine (DICOM) format from the Picture Archiving and Communication System (PACS). Specifically, the venous phase scans for each patient were identified and subsequently converted to the Neuroimaging Informatics Technology Initiative (NIfTI) format to facilitate subsequent 3D processing. Prior to further processing, a window level ranging from − 50 to 250 HU was selected. During the training process, we augmented the image dataset by applying techniques such as translation, rotation, scaling, and elastic distortions, thereby increasing the diversity and variability of the training samples.

**Fig. 2:**
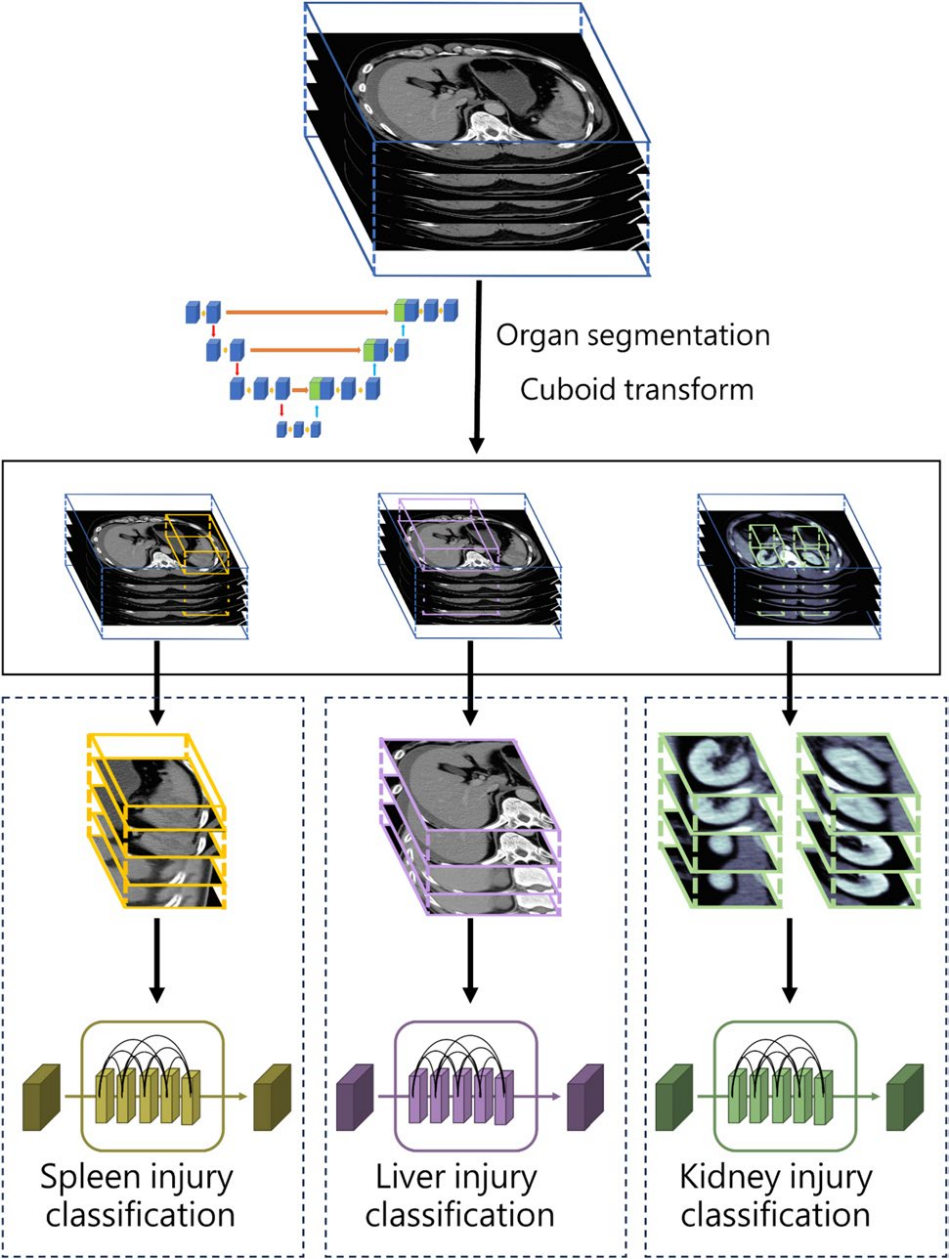
A comprehensive overview of the algorithm design for solid organ injury detection

We designed a two-step DL algorithm to detect specific solid organ injuries, as demonstrated in Fig. 2. First, to reduce the labeling effort, we apply an open-access organ segmentation model, Totalsegmentator v.1.3 [39], to generate the solid organ segmentation masks, including the spleen, liver, and kidney. The generated mask was then transformed into a 3D cuboid box to include the surrounding background of the target organ. The 3D cuboid box of each organ in the development dataset was used to train the injury classification network. All images were resized to 64 × 64 × 64 before fitting into the classification model.

### Solid Organ Injury Classification Network

The solid organ injury classification model was trained using entire abdominal CT scans as a baseline to compare with individual two-step organ injury classification models. The individual organ model was trained using the cropped cuboid image generated by the organ detection model. The spleen, liver, and kidney injury classification model was trained separately. The cropped organ was fed into a 3D Convolutional Block Attention Module (CBAM) neural network [41] with a binary classification label. The input has dimensions of 64 × 64 × 64. Initially, it is processed through two 3D convolutional layers, generating outputs with four channels. Following this, the input goes through three distinct blocks, each comprising a varying number of residual blocks with a CBAM integrated as the final layer in each block. The process is completed with a Global Average Pooling and a Fully Connected Network layer. The final output dimensions are 8 × 8 × 8. Ultimately, the entire network is trained using the angular softmax loss, which facilitates the learning of features that are discriminative in terms of their angular properties for classification. For example, when training the spleen injury classification model, we only identify whether the spleen is injured despite other intra-abdominal organ injuries to eliminate the interference of other organs. We use the grad-CAM algorithm [42] to visualize whether the model focused on the target lesion to determine the reliability of the result. The results of the three organ model are also combined to calculate an overall solid organ injury detection rate.

### Software and Statistical Analysis

The experimental setup utilized a workstation equipped with an Intel(R) Core(TM) i9-10900X CPU operating at 3.70 GHz, accompanied by 96 GB RAM, and NVIDIA TITAN RTX and GeForce RTX 3090 GPUs. The workstation ran on an Ubuntu 18.04 operating system. The entire pipeline was implemented using Python v3.6.9 and PyTorch v1.6.0. Preprocessing of the images involved employing various Python libraries such as diocom2nifti, NiBabel, SciPy, and OpenCV. The image annotation process was conducted using the Medical Imaging Interaction Toolkit (MITK). At the same time, data augmentation was performed utilizing the tools provided by the Medical Open Network for Artificial Intelligence (MONAI).

**Fig. 3:**
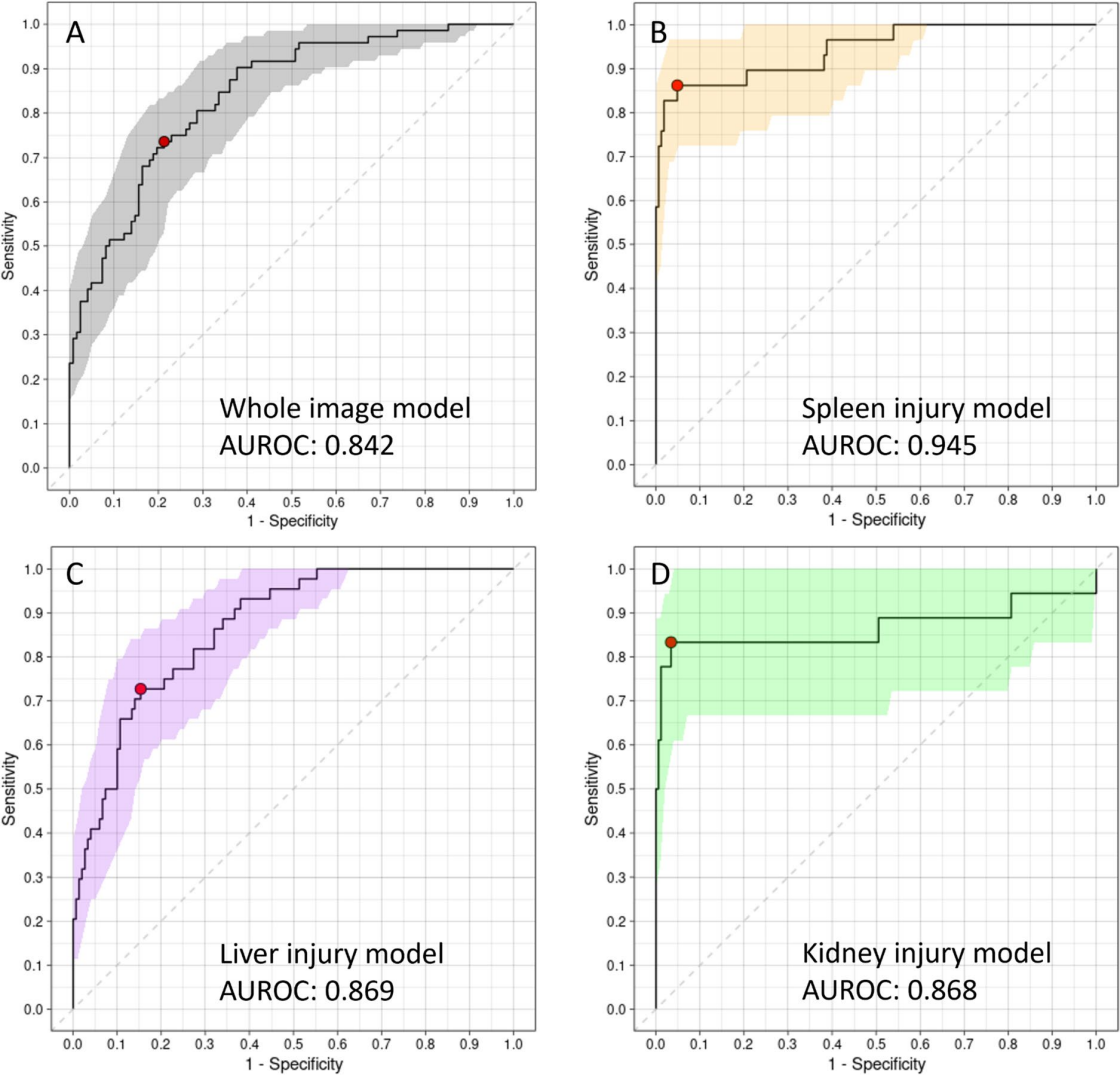
The ROC curve and AUROC of each solid organ injury detection model. **A** Whole image model. **B** Spleen injury model. **C** Liver injury model. **D** Kidney injury model. The shaded area represented the 95% confidence interval

For the statistical analysis, we utilized R version 4.2.2 with the “pROC” package. Model classification performance was evaluated through a confusion matrix, allowing us to assess accuracy, sensitivity, specificity, false positive rate (FPR), false negative rate (FNR), positive predictive value (PPV), and negative predictive value (NPV). Furthermore, we employed the receiver operating characteristic (ROC) curve and calculated the area under the ROC curve (AUROC) to assess model performance (Fig. 3). The optimal cutoff value was determined using the Youden index, and all model performances were compared based on the cutoff value with the best Youden index. To estimate the confidence interval of the ROC curve, we utilized the bootstrapping method. The comparisons of performance metrics between each organ-specific model and the whole image model were conducted using McNemar’s test or the binomial proportions test, as appropriate. The continuous variables of the demographic data were compared using the Kruskal–Wallis rank-sum test, while categorical variables were compared using the chi-square test.

## Results

We gathered a total of 1496 venous phase abdominal CT scans from an equal number of patients. From this dataset, we preserved 194 scans acquired in the last year as an independent test set, while the remaining 1302 scans were allocated to form the development dataset. Table 1 displays the demographic characteristics of this dataset. To ensure improved algorithm training, we balanced the classes within the development set. In the test set, the proportion of positive cases is relatively small, reflecting the clinical distribution. Among the 72 positive cases identified, 16 patients (22.2%) were found to have more than one solid organ injury.

**Table 1:** Demographic characteristics of the dataset

	Development set (*n* = 1302)	Test set (n = 194)	*p* value
Age, median [IQR]	44.00 [25.00, 69.00]	51.50 [28.00, 72.00]	0.033
Gender (male), *n* (%)	873 (67.1)	132 (68.0)	0.848
Etiology			0.003
Motor vehicle accident, *n* (%)	727 (55.8)	88 (45.4)	
Fall, *n* (%)	160 (12.3)	22 (11.3)	
Mechanical injury, *n* (%)	727 (55.8)	88 (45.4)	
Other mechanisms, *n* (%)	47 (3.6)	7 (3.6)	
Acute abdomen, *n* (%)	325 (25.0)	74 (38.1)	
ISS, median [IQR]	18.00 [9.00, 29.00]	17.00 [9.00, 26.00]	0.983
NISS, median [IQR]	22.00 [12.00, 29.00]	22.00 [13.00, 34.00]	0.478
Solid organ injury, *n* (%)	668 (51.3)	72 (37.1)	< 0.001
Spleen OIS, *n* (%)			0.082
Negative	1026 (78.8)	165 (85.1)	
Grade 1	39 (3.0)	2 (1.0)	
Grade 2	82 (6.3)	5 (2.6)	
Grade 3	56 (4.3)	10 (5.2)	
Grade 4	70 (5.4)	6 (3.1)	
Grade 5	29 (2.2)	6 (3.1)	
Liver OIS, *n* (%)			0.818
Negative	969 (74.4)	150 (77.3)	
Grade 1	71 (5.5)	6 (3.1)	
Grade 2	110 (8.4)	17 (8.8)	
Grade 3	96 (7.4)	13 (6.7)	
Grade 4	47 (3.6)	7 (3.6)	
Grade 5	9 (0.7)	1 (0.5)	
Kidney OIS, *n* (%)			0.114
Negative	1086 (83.4)	176 (90.7)	
Grade 1	71 (5.5)	4 (2.1)	
Grade 2	62 (4.8)	5 (2.6)	
Grade 3	41 (3.1)	3 (1.5)	
Grade 4	28 (2.2)	5 (2.6)	
Grade 5	14 (1.1)	1 (0.5)	

The baseline whole image model exhibited a reasonably good AUROC of 0.842; however, it lacked the capability to identify the specific injured organ accurately. Even the visualization heatmap was unsuccessful in pinpointing the site of injury. Among the 72 patients with solid organ injuries, 19 (26.4%) cases were missed by this model. On the other hand, the spleen injury classification model displayed a high accuracy of 0.938 and successfully identified 25 (86.2% sensitivity) of the spleen-injured patients with eight false positives (5% FPR). The four patients the model missed were all cases of low-grade splenic injury. Similarly, the liver injury model showed a slight improvement over the whole image model, achieving an AUROC of 0.869. Nonetheless, it still failed to identify 12 patients with liver injuries (16.7% FNR), with 23 false positives(15% FPR). For the kidney model, evaluation on both sides of the kidneys demonstrated a high specificity of 96.6% (6 false positives, 3.4% FPR), but the sensitivity was relatively low at 83.3% (6 false negatives, 33.3 FNR). Combining the three organ models, the overall accuracy, sensitivity, and specificity to detect solid organ injuries in the CT scan reached 84.0%, 87.5%, and 82.0%, respectively (Table 2).

**Table 2:** Performance of solid organ injury detection models on the test set

	Whole image model	Spleen injury model	*p* value	Liver injury model	*p* value	Kidney injury model	*p* value	Combined model	*p* value
AUROC (95% CI)	0.841 (0.781–0.895)	0.945 (0.888–0.988)	0.008	0.868 (0.811–0.918)	0.499	0.870 (0.721–0.994)	0.7344	–	–
Accuracy (95% CI)	0.768 (0.706–0.825)	0.938 (0.902–0.969)	< 0.05	0.820 (0.768–0.876)	0.259	0.954 (0.923–0.979)	< 0.05	0.840 (0.781–0.889)	0.655
Sensitivity (95% CI)	0.736 (0.625–0.833)	0.862 (0.724–0.966)	0.270	0.727 (0.591–0.864)	1.000	0.833 (0.667–1.000)	0.581	0.875 (0.799–0.951)	0.041
Specificity (95% CI)	0.787 (0.713–0.861)	0.952 (0.915–0.982)	< 0.05	0.847 (0.793–0.907)	0.264	0.966 (0.938–0.989)	< 0.05	0.820 (0.751–0.888)	0.480
PPV (95% CI)	0.671 (0.591–0.759)	0.758 (0.632–0.893)	0.494	0.583 (0.490–0.696)	0.384	0.714 (0.560–0.889)	0.908	0.741 (0.648–0.834)	0.177
NPV (95% CI)	0.835 (0.780–0.891)	0.975 (0.951–0.994)	< 0.05	0.915 (0.876–0.954)	0.086	0.983 (0.965–1.000)	< 0.05	0.917 (0.866–0.969)	0.031

The *p* value of each model was statistically analyzed compared with the whole image model.

## Discussion

**Fig. 4:**
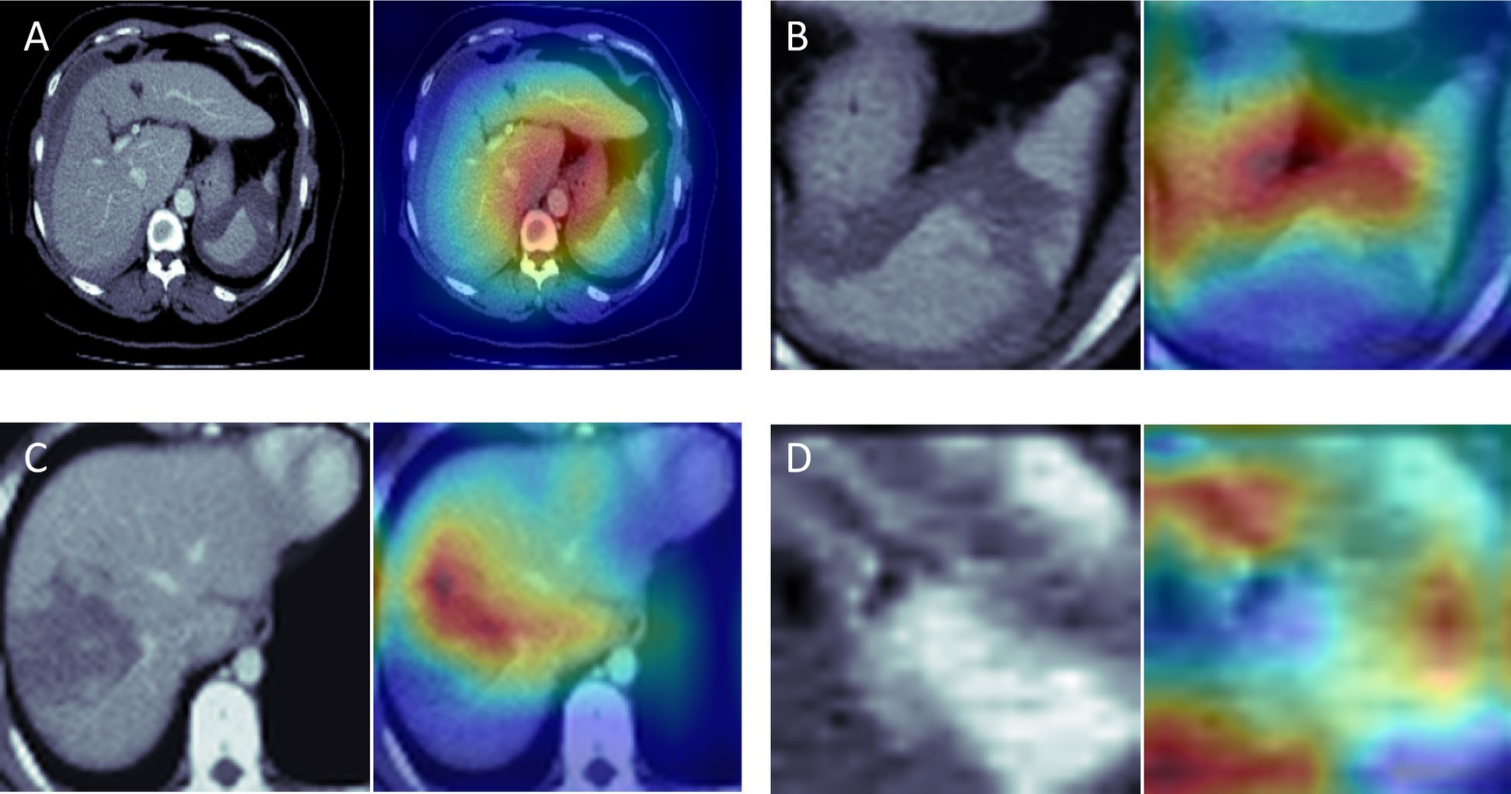
Visualization examples of each solid organ injury detection model. **A** The heatmap from the whole image model indicates a failure to localize the spleen injury. **B** The heatmap from the spleen injury model accurately highlights the lacerated area and hematoma surrounding the spleen. **C** The heatmap of the liver injury model successfully localizes a grade 3 injury. **D** The heatmap points out the laceration in the kidney and the presence of a perirenal hematoma

Up until now, we have developed a DL algorithm that automates the detection of solid organ injuries in abdominal CT scans. The diagnostic accuracy achieved 0.938 (0.902–0.969), 0.820 (0.763–0.871), and 0.959 (0.933–0.979) for splenic, hepatic, and renal injuries, respectively. These accuracy levels are accompanied by satisfactory sensitivity and specificity. Notably, for splenic and renal injuries, the model showed good diagnostic accuracy, providing precise locations of injuries through the use of heatmaps. The accuracy for hepatic injuries is comparatively lower, possibly owing to the cuboid cropping method, which includes neighboring organs and introduces noise during model training, thereby diminishing performance. However, the diagnostic accuracy remains acceptable. This study is the first to introduce a multitask DL model specifically designed for trauma detection in abdominal CT scans. To enhance the model’s explainability, we have incorporated Grad-CAM producing heatmaps into the examined images. This technique is commonly applied in medical image analysis to visualize the dominant parts of the input image for the prediction [43]. As in Fig. 4, the heatmaps focused on the injured organ part in our test set; however, this feature can only demonstrate the possible area the model focused on to make the decision rather than precisely contouring the injured area. Segmenting the injured part requires a huge labeling effort, especially for the ambitious boundary of the current task, which is challenging future work. The average processing time to generate results in our hardware setting was only 3 min, and the process can be automatically initiated after the completion of image acquisition. In the context of trauma treatment, where time is critical, reducing diagnostic time can lead to early detection and intervention, potentially minimizing the risk of significant blood loss and improving patient outcomes.

The shift from operative to nonoperative management in patients with BAT has been primarily driven by advancements in diagnostic tools and a reduction in complications associated with operative procedures [8, 44]. The critical and essential step in this process is the careful selection of suitable patients. Abdominal CT scans play a vital role in assisting traumatologists with patient selection [45–47]. By utilizing DLM support, we can achieve a high negative predictive rate, with values of 0.975 (0.952–0.994), 0.914 (0.876–0.949), and 0.967 (0.945–0.989) for splenic, hepatic, and renal injuries, respectively. A secondary check by clinical experts can further reduce the misdiagnosis rate. The ultimate goal for both clinical data scientists and clinicians is to develop a multitask DLM that can significantly accelerate the patient evaluation process [48]. By leveraging the concepts demonstrated in this study, the future of solid organ injury detection modeling holds tremendous potential. The combination of advanced diagnostic tools and DLM support is paving the way for more accurate and efficient patient assessments, ultimately leading to improved trauma care outcomes.

Detecting traumatic solid organ injuries poses a significant challenge in algorithm development, primarily due to the complex nature of image morphology and the occurrence of multiple organ injuries simultaneously. Often, injured organs share similar radiological findings, such as hemoperitoneum, hematoma, or contrast extravasation, making it difficult to distinguish between them. Moreover, accurately defining the precise location of the injured part within a specific organ proves to be a daunting task, particularly when compared with other body parts like brain hemorrhage identification [49]. In our current study, we have devised a two-step algorithm design to address these challenges effectively. By focusing on specific organs in the first step, we aim to reduce the complexity and improve the algorithm’s accuracy in detecting organ-specific injuries. Additionally, to enhance the interpretability of the model, we employ a heatmap localization technique in the second step, allowing us to highlight the injured region within the specific organ. This localization approach greatly enhances the model’s explainability, facilitating better understanding and trust in the detection results. Through these advancements, our algorithm demonstrates promising results in detecting traumatic solid organ injuries, offering potential benefits for clinical applications. A follow-up study in the future focusing on the expert evaluation of heatmaps could provide a deeper, clinically relevant understanding of our model’s utility in medical diagnostics.

Previous research has concentrated on segmentation and the automatic grading of the severity of injured organs. Drezin et al. utilized a DL-based approach for segmentation, enhanced by decision tree analysis, to predict significant arterial damage in liver trauma, achieving an accuracy of 0.84 [25]. Similarly, Farzaneh et al. proposed a framework for liver trauma detection and quantitative assessment applied to 77 CT scans [50]. Chen et al. developed a four-component algorithm for the automatic grading of spleen injuries [51], and Farzaneh et al. also described automated kidney segmentation for trauma patients using active contour modeling [52]. Zhou et al. use an external attention and synthetic phase augmentation module on a small dataset to improve the multiphase splenic vascular injury segmentation with DeepLab-v3 baseline [33].

Additionally, Tulum et al. developed a computer-aided segmentation system for traumatic kidney analysis [53]. In the classification realm for detecting organ injury, Wang et al. employed machine learning techniques with 3D active contours to identify spleen injuries, using a dataset of 54 healthy and 45 lacerated spleens. This method was validated with fivefold cross-validation, achieving an AUC of 0.91 in the test set [32]. Hamghalam et al. developed a DL model using 608 scans of each of the injured and noninjured spleens, achieving an accuracy of 0.808 with fivefold cross-validation [54]. Compared with the previous studies, our approach offers an alternative approach to multitask DL algorithm design. We can obtain information about multiple solid organ injuries by inputting CT images into our model instead of focusing on a single organ. This design is particularly well suited for the high-tension and crowded environment of trauma bays. Developing a globalized or generalized algorithm to address all issues within the same images can be challenging, especially with limited resources. However, with careful consideration of clinical domain knowledge and well-defined data labeling, it is possible to develop clinically beneficial algorithms tailored to specific problems in medical image analysis. Large high-technology companies have also shown interest in these sectors [55, 56], and their involvement is expected to drive significant improvements in this technology. A notable advantage of the current algorithm trends is the problem-oriented approach, where algorithms are tailored to address specific medical challenges.

Moreover, using automatically cropped images and advanced analysis has the added benefit of reducing calculation time and lightening the workload on workstations. Although the application of the Totalsegmentator will make the inference time longer, the cropping step can reduce the noise from the surrounding organ for classification tasks to improve performance. This, in turn, translates to cost savings, as the investment and equipment requirements for deploying the AI algorithm can be kept reasonable. While DL algorithms have proven their potential in assisting various healthcare tasks, little attention has been given to discussing the costs and minimal computer system requirements. Innovative DL network architectures, such as Transformer-based structures, have the potential to deliver superior performance and eliminate the need for the cropping step. However, this approach requires significantly more computational power, particularly for processing 3D images. Setting up a system capable of deploying DL algorithms often necessitates additional servers or computers equipped with graph processing units (GPUs), which can increase expenses for institutes. To address this, efforts have been made to optimize GPU usage and minimize the necessity of high-end computers to make DL algorithms more accessible and cost-effective for implementation.

## Limitations

The current study has several limitations. Firstly, the algorithm was trained using images from a single trauma center, raising concerns about its performance when applied to images from other institutions with varying CT protocols and image acquisition methods. This discrepancy could affect the algorithm’s generalizability and raise questions about its reliability in different clinical settings. Secondly, the ground truth is based on the radiologist’s report and a single annotator’s confirmation. Moreover, the imbalanced distribution of solid organ injuries, such as kidney injury being relatively rare compared to spleen and liver injuries, may introduce bias during the performance evaluation. This could potentially lead to an overestimation of the algorithm’s accuracy for more common injuries and an underestimation for less frequent ones. The segmentation mask generated by the Totalsegmentator is also a concern for those largely deformed organs. This can lead to failure on the cropping step since the Totalsegmentator is trained on nontrauma images. Prospective multicenter data collection, incorporating clinical trauma scenario class distribution, is imperative to ensure the robustness of the algorithm and to address these limitations. Incorporating multiple independent trauma specialist radiologists for image annotation in future studies promises to not only enhance lesion localization and characterization accuracy but also to address the issue of inconsistent AAST grading agreement [57]. Conducting an evaluation on a diverse dataset from multiple centers with a graphic user interface will help verify the algorithm’s performance in real-world scenarios, accounting for various imaging protocols and organ injury distributions. It will strengthen the credibility and applicability of the algorithm as a clinical tool.

## Conclusion

The developed DLM serves as a valuable tool to assist medical professionals in identifying traumatic solid organ injuries with promising diagnostic accuracy. It is essential to note that the algorithm does not aim to replace the expertise and judgment of clinicians; instead, it complements their skills and knowledge. By leveraging the DLM, medical professionals can use this tool to accelerate the diagnostic process and improve the overall efficiency of trauma care.

## Data Availability

The image dataset is not publicly accessible but can be obtained upon submission of a justified request and subsequent approval by the Institutional Review Board of Chang Gung Medical Foundation in alignment with the institution's policies. Conversely, the source code employed in this study is accessible and can be found at the following URL: https://github.com/houhsein/ABD_solid_organ_injury_detection.

## References

[1] Mehta N, Babu S, Venugopal K (2014). An experience with blunt abdominal trauma: evaluation, management and outcome. Clin Pract.

[2] Hsieh T-M, Cheng Tsai T, Liang J-L, Che Lin C (2014). Non-operative management attempted for selective high grade blunt hepatosplenic trauma is a feasible strategy. World J Emerg Surg.

[3] Kendall JL, Kestler AM, Whitaker KT, Adkisson M-M, Haukoos JS (2011). Blunt abdominal trauma patients are at very low risk for intra-abdominal injury after emergency department observation. West J Emerg Med.

[4] Wiik Larsen J, Søreide K, Søreide JA, Tjosevik K, Kvaløy JT, Thorsen K (2022). Epidemiology of abdominal trauma: An age- and sex-adjusted incidence analysis with mortality patterns. Injury.

[5] Stassen NA, Bhullar I, Cheng JD, Crandall M, Friese R, Guillamondegui O (2012). Nonoperative management of blunt hepatic injury: an Eastern Association for the Surgery of Trauma practice management guideline. J Trauma Acute Care Surg.

[6] Stassen NA, Bhullar I, Cheng JD, Crandall ML, Friese RS, Guillamondegui OD (2012). Selective nonoperative management of blunt splenic injury: an Eastern Association for the Surgery of Trauma practice management guideline. J Trauma Acute Care Surg.

[7] Altman AL, Haas C, Dinchman KH, Spirnak JP. Selective nonoperative management of blunt grade 5 renal injury. J Urol 2000;164:27–30; discussion 30–1. 10840417

[8] Raza M, Abbas Y, Devi V, Prasad KVS, Rizk KN, Nair PP. Non operative management of abdominal trauma – a 10 years review. World J Emerg Surg 2013;8:14. 10.1186/1749-7922-8-14. 10.1186/1749-7922-8-14 PMC3636075 23561288

[9] Achatz G, Schwabe K, Brill S, Zischek C, Schmidt R, Friemert B (2022). Correction to: Diagnostic options for blunt abdominal trauma. Eur J Trauma Emerg Surg.

[10] Stuhlfaut JW, Anderson SW, Soto JA (2007). Blunt abdominal trauma: current imaging techniques and CT findings in patients with solid organ, bowel, and mesenteric injury. Semin Ultrasound CT MR.

[11] Ingram M-CE, Siddharthan RV, Morris AD, Hill SJ, Travers CD, McKracken CE, et al. Hepatic and splenic blush on computed tomography in children following blunt abdominal trauma: Is intervention necessary? J Trauma Acute Care Surg 2016;81:266–70. 10.1097/TA.0000000000001114. 10.1097/TA.0000000000001114 27257698

[12] Gamanagatti S, Rangarajan K, Kumar A, Jineesh. Blunt abdominal trauma: imaging and intervention. Curr Probl Diagn Radiol 2015;44:321–36. 10.1067/j.cpradiol.2015.02.005. 10.1067/j.cpradiol.2015.02.005 25801463

[13] Mahmood I, Tawfek Z, Abdelrahman Y, Siddiuqqi T, Abdelrahman H, El-Menyar A (2014). Significance of computed tomography finding of intra-abdominal free fluid without solid organ injury after blunt abdominal trauma: time for laparotomy on demand. World J Surg.

[14] Yanar H, Ertekin C, Taviloglu K, Kabay B, Bakkaloglu H, Guloglu R (2008). Nonoperative treatment of multiple intra-abdominal solid organ injury after blunt abdominal trauma. J Trauma.

[15] El-Menyar A, Abdelrahman H, Al-Hassani A, Peralta R, AbdelAziz H, Latifi R (2017). Single Versus Multiple Solid Organ Injuries Following Blunt Abdominal Trauma. World J Surg.

[16] Safavi A, Skarsgard ED, Rhee P, Zangbar B, Kulvatunyou N, Tang A (2016). Trauma center variation in the management of pediatric patients with blunt abdominal solid organ injury: a national trauma data bank analysis. J Pediatr Surg.

[17] Cimbanassi S, Chiara O, Leppaniemi A, Henry S, Scalea TM, Shanmuganathan K (2018). Nonoperative management of abdominal solid-organ injuries following blunt trauma in adults: Results from an International Consensus Conference. J Trauma Acute Care Surg.

[18] van Schuppen J, Olthof DC, Wilde JCH, Beenen LFM, van Rijn RR, Goslings JC (2014). Diagnostic accuracy of a step-up imaging strategy in pediatric patients with blunt abdominal trauma. Eur J Radiol.

[19] Pinto A, Reginelli A, Pinto F, Lo Re G, Midiri F, Muzj C (2016). Errors in imaging patients in the emergency setting. Br J Radiol.

[20] Liu X, Faes L, Kale AU, Wagner SK, Fu DJ, Bruynseels A (2019). A comparison of deep learning performance against health-care professionals in detecting diseases from medical imaging: a systematic review and meta-analysis. Lancet Digit Health.

[21] Cheng C-T, Wang Y, Chen H-W, Hsiao P-M, Yeh C-N, Hsieh C-H (2021). A scalable physician-level deep learning algorithm detects universal trauma on pelvic radiographs. Nat Commun.

[22] Choi J, Mavrommati K, Li NY, Patil A, Chen K, Hindin DI (2022). Scalable deep learning algorithm to compute percent pulmonary contusion among patients with rib fractures. J Trauma Acute Care Surg.

[23] Avendi MR, Kheradvar A, Jafarkhani H (2016). A combined deep-learning and deformable-model approach to fully automatic segmentation of the left ventricle in cardiac MRI. Med Image Anal.

[24] Liu J, Varghese B, Taravat F, Eibschutz LS, Gholamrezanezhad A. An Extra Set of Intelligent Eyes: Application of Artificial Intelligence in Imaging of Abdominopelvic Pathologies in Emergency Radiology. Diagnostics (Basel) 2022;12. 10.3390/diagnostics12061351. 10.3390/diagnostics12061351 PMC9221728 35741161

[25] Dreizin D, Chen T, Liang Y, Zhou Y, Paes F, Wang Y (2021). Added value of deep learning-based liver parenchymal CT volumetry for predicting major arterial injury after blunt hepatic trauma: a decision tree analysis. Abdom Radiol (NY).

[26] Chiu I-M, Lin C-HR, Yau F-FF, Cheng F-J, Pan H-Y, Lin X-H, et al. Use of a Deep-Learning Algorithm to Guide Novices in Performing Focused Assessment With Sonography in Trauma. JAMA Netw Open 2023;6:e235102. 10.1001/jamanetworkopen.2023.5102. 10.1001/jamanetworkopen.2023.5102 PMC10051044 36976564

[27] Choi J, Alawa J, Tennakoon L, Forrester JD (2022). DeepBackRib: Deep learning to understand factors associated with readmissions after rib fractures. J Trauma Acute Care Surg.

[28] Dreizin D, Staziaki PV, Khatri GD, Beckmann NM, Feng Z, Liang Y (2023). Artificial intelligence CAD tools in trauma imaging: a scoping review from the American Society of Emergency Radiology (ASER) AI/ML Expert Panel. Emerg Radiol.

[29] Agrawal A, Khatri GD, Khurana B, Sodickson AD, Liang Y, Dreizin D (2023). A survey of ASER members on artificial intelligence in emergency radiology: trends, perceptions, and expectations. Emerg Radiol.

[30] Harris RJ, Kim S, Lohr J, Towey S, Velichkovich Z, Kabachenko T (2019). Classification of Aortic Dissection and Rupture on Post-contrast CT Images Using a Convolutional Neural Network. J Digit Imaging.

[31] Huang S, Zhou Z, Qian X, Li D, Guo W, Dai Y (2022). Automated quantitative assessment of pediatric blunt hepatic trauma by deep learning-based CT volumetry. Eur J Med Res.

[32] Wang J, Wood A, Gao C, Najarian K, Gryak J. Automated Spleen Injury Detection Using 3D Active Contours and Machine Learning. Entropy 2021;23. 10.3390/e23040382. 10.3390/e23040382 PMC8063804 33804831

[33] Zhou Y, Dreizin D, Wang Y, Liu F, Shen W, Yuille AL (2022). External Attention Assisted Multi-Phase Splenic Vascular Injury Segmentation With Limited Data. IEEE Trans Med Imaging.

[34] Dreizin D, Zhou Y, Chen T, Li G, Yuille AL, McLenithan A (2020). Deep learning-based quantitative visualization and measurement of extraperitoneal hematoma volumes in patients with pelvic fractures: Potential role in personalized forecasting and decision support. J Trauma Acute Care Surg.

[35] Dreizin D, Nixon B, Hu J, Albert B, Yan C, Yang G (2022). A pilot study of deep learning-based CT volumetry for traumatic hemothorax. Emerg Radiol.

[36] Dreizin D, Zhou Y, Fu S, Wang Y, Li G, Champ K (2020). A Multiscale Deep Learning Method for Quantitative Visualization of Traumatic Hemoperitoneum at CT: Assessment of Feasibility and Comparison with Subjective Categorical Estimation. Radiol Artif Intell.

[37] Dreizin D, Zhou Y, Zhang Y, Tirada N, Yuille AL (2020). Performance of a Deep Learning Algorithm for Automated Segmentation and Quantification of Traumatic Pelvic Hematomas on CT. J Digit Imaging.

[38] Cheng C-T, Lin H-S, Hsu C-P, Chen H-W, Huang J-F, Fu C-Y (2023). The three-dimensional weakly supervised deep learning algorithm for traumatic splenic injury detection and sequential localization: an experimental study. Int J Surg.

[39] Wasserthal J, Meyer M, Breit H-C, Cyriac J, Yang S, Segeroth M. TotalSegmentator: robust segmentation of 104 anatomical structures in CT images. arXiv [eessIV] 2022. 10.1148/ryai.230024 PMC10546353 37795137

[40] Kozar RA, Crandall M, Shanmuganathan K, Zarzaur BL, Coburn M, Cribari C (2018). Organ injury scaling 2018 update: Spleen, liver, and kidney. J Trauma Acute Care Surg.

[41] Woo S, Park J, Lee J-Y, Kweon IS. CBAM: Convolutional Block Attention Module. Computer Vision – ECCV 2018, Cham: Springer International Publishing; 2018, p. 3–19. 10.1007/978-3-030-01234-2_1.

[42] Selvaraju RR, Cogswell M, Das A, Vedantam R, Parikh D, Batra D. Grad-cam: Visual explanations from deep networks via gradient-based localization. Proceedings of the IEEE international conference on computer vision, 2017, p. 618–26.

[43] Salahuddin Z, Woodruff HC, Chatterjee A, Lambin P (2022). Transparency of deep neural networks for medical image analysis: A review of interpretability methods. Comput Biol Med.

[44] Malhotra AK, Fabian TC, Croce MA, Gavin TJ, Kudsk KA, Minard G (2000). Blunt hepatic injury: a paradigm shift from operative to nonoperative management in the 1990s. Ann Surg.

[45] Coccolini F, Coimbra R, Ordonez C, Kluger Y, Vega F, Moore EE (2020). Liver trauma: WSES 2020 guidelines. World J Emerg Surg.

[46] Sharma OP, Oswanski MF, Singer D (2005). Role of repeat computerized tomography in nonoperative management of solid organ trauma. Am Surg.

[47] Lee JT, Slade E, Uyeda J, Steenburg SD, Chong ST, Tsai R (2021). American Society of Emergency Radiology Multicenter Blunt Splenic Trauma Study: CT and Clinical Findings. Radiology.

[48] Zhao Y, Wang X, Che T, Bao G, Li S (2023). Multi-task deep learning for medical image computing and analysis: A review. Comput Biol Med.

[49] Monteiro M, Newcombe VFJ, Mathieu F, Adatia K, Kamnitsas K, Ferrante E (2020). Multiclass semantic segmentation and quantification of traumatic brain injury lesions on head CT using deep learning: an algorithm development and multicentre validation study. The Lancet Digital Health.

[50] Farzaneh N, Stein EB, Soroushmehr R, Gryak J, Najarian K (2022). A deep learning framework for automated detection and quantitative assessment of liver trauma. BMC Med Imaging.

[51] Chen H, Unberath M, Dreizin D (2023). Toward automated interpretable AAST grading for blunt splenic injury. Emerg Radiol.

[52] Farzaneh N, Reza Soroushmehr SM, Patel H, Wood A, Gryak J, Fessell D (2018). Automated Kidney Segmentation for Traumatic Injured Patients through Ensemble Learning and Active Contour Modeling. Conf Proc IEEE Eng Med Biol Soc.

[53] Tulum G, Teomete U, Cuce F, Ergin T, Koksal M, Dandin O (2019). Automated segmentation of the injured kidney due to abdominal trauma. J Med Syst.

[54] Hamghalam M, Moreland R, Gomez D, Simpson A, Lin HM, Jandaghi AB, et al. Machine Learning Detection and Characterization of Splenic Injuries on Abdominal Computed Tomography. Can Assoc Radiol J 2024:8465371231221052. 10.1177/08465371231221052. 10.1177/08465371231221052 38189316

[55] Esteva A, Chou K, Yeung S, Naik N, Madani A, Mottaghi A (2021). Deep learning-enabled medical computer vision. NPJ Digit Med.

[56] Egger J, Gsaxner C, Pepe A, Pomykala KL, Jonske F, Kurz M (2022). Medical deep learning—A systematic meta-review. Comput Methods Programs Biomed.

[57] Adams-McGavin RC, Tafur M, Vlachou PA, Wu M, Brassil M, Crivellaro P, et al. Interrater Agreement of CT Grading of Blunt Splenic Injuries: Does the AAST Grading Need to Be Reimagined? Can Assoc Radiol J 2023:8465371231184425. 10.1177/08465371231184425. 10.1177/08465371231184425 37405424

